# The efficacy of intensified sensory therapy on upper extremity functions and activities of daily living in patients with chronic stroke: A randomized controlled trial

**DOI:** 10.12669/pjms.42.1.11940

**Published:** 2026-01

**Authors:** Muhammed Rohat Yazici, Cigdem Cekmece

**Affiliations:** 1 Muhammed Rohat Yazici, PhD Degree Student. Department of Occupational Therapy, Uskudar University, Istanbul, Turkey; 2 Cigdem Cekmece, OT, PhD. Assistant of Professor, Section of Occupational Therapy, Department of Therapy and Rehabilitation, Vocational School of Kocaeli Health Services, Kocaeli University, Kocaeli, Turkey

**Keywords:** Occupational Therapy, Stroke, Sensory Therapy

## Abstract

**Objective:**

The aim of this study was to investigate the efficacy of intensified sensory therapy on upper extremity functions, daily activity and life quality of stroke survivors (SS).

**Methodology:**

This randomized controlled trial included 30 chronic SS (18 males - 12 females) who were treated at Kocaeli University Hospital, Department of Physical Medicine and Rehabilitation between May 2022 and September 2022. Participants were randomly assigned to a Sensory-Training Group (STG; n = 15; mean age= 59.07±12.73 years) and a Control Group (CG; n = 15; mean age= 56.53±13.80 years). Both groups received 15 sessions of 30 minutes of physical therapy, occupational therapy (OT), and 20 minutes of activity daily living (ADL) training for three weeks. The STG also received intensified sensory therapy during each session. Thumb localization, finger shift, and stereognosis tests were performed in both groups only before the start of treatment. The Jebsen Taylor Hand Function Test (JTHFT), Modified Frenchay Scale (MFS), Canadian Occupational Performance Measure (COPM), Goal Attainment Scale (GAS), and Stroke Specific Quality of Life Scale (SS-QOL) were administered before and after treatment.

**Results:**

A statistically significant difference was found between STG and CG in all parameters of the MFS, JTHFT, COPM (performance and satisfaction), GAS, and all parameters of SS-QOL except for the language, thinking, and seeing parameters.

**Conclusion:**

These results suggest that sensory therapies applied with conventional treatments increased upper extremity functions, ADL performance, participation rate and quality of life of the SS.

***Clinical Trials Registry:*** NCT05133219.

## INTRODUCTION

Sensory deficits are one of the most common complications of stroke, and the prevalence of these problems is between 11% and 85% in individuals who have had a stroke.^1^ In up to 85% of people affected by stroke, sensory disorders in the upper extremity, characterized by a decreased sense of touch, temperature, pain, and proprioception have been observed.^2^ Sensory loss significantly influences functional recovery, particularly impacting reaching, dexterity, and inter-limb coordination.^3^ Sensory inputs play a fundamental role in motor recovery after a stroke. It is known that any motor movement needs the integration of varying degrees of sensory information and thus part of any motor training rehabilitation also should include sensory training.^4^

In published studies, different forms of somatosensory stimulation performed within the scope of sensory therapy have been shown to facilitate motor behaviors.^4^-^6^ However, much less attention is paid to treating sensory disorders in stroke rehabilitation. Instead, previous studies generally focused on motor functions, exercises, and activities for the upper/lower extremities.^7^,^8^ Despite increasing evidence that specific sensory training may be beneficial, applying the training approach in clinical practice has yet to become widespread.^4^,^6^ This randomized controlled trial aimed to examine the effects of intensive sensory therapy on upper limb function, participation, activities of daily living (ADL) and quality of life in stroke survivors (SS).

## METHODOLOGY

This study included 30 SS who received treatment at a University Hospital Department of Physical and Rehabilitation Medicine from May to September 2022. The inclusion criteria were aged between 18 and 80 years, medically stable, a post-stroke interval of at least six months. SS were not considered for inclusion in this study if they exhibited severe spasticity, indicated by a Modified Ashworth Scale (MAS) score of three or higher in upper extremity muscles, had joint limitations (contractures), congestive heart failure, peripheral arterial disease, severe dementia, language impairments, or experienced highly painful conditions like reflex sympathetic dystrophy (RSD). All participants provided informed, written consent before participating in the study.

### Ethical Approval and Clinical Trials Registry:

This study was approved by the University Ethical Committee (KAEK 2021/04.39) and was registered with the Clinical Trials Registry (NCT05133219).

Demographic information and sensory evaluation (Thumb Localization Test, Finger Slide Test and Stereognosis Test) results of SS were recorded before treatment. At the beginning and end of the treatment, the Jebsen Taylor Hand Function Test (JTHFT), Modified Frenchay Scale (MFS), Canadian Occupational Performance Measurement (COPM), Goal Attainment Scaling (GAS), and Stroke-Specific Quality of Life Scale (SS-QOL), were applied. To ensure standardization, all evaluations were made by the same researcher who was blind to the treatment group. SS were randomly assigned to either receive sensory therapy in addition to conventional therapies or not. All SS in both groups were included in the physical therapy, occupational therapy (OT), and activities daily living training program over three weeks for five days a week. Sensory therapy was applied to the sensory-training group (STG) in addition to these treatments.

### Data analysis:

Statistical analysis was done with IBM SPSS, version 20.0 (IBM Corp, Armonk, NY, USA). Normality of data set distribution was evaluated with the Shapiro-Wilk Test. Normally distributed numerical variables are given as mean±standard deviation, non-normally distributed numerical variables as median (25th-75th percentile), and categorical variables as frequency (percentage). The difference between the groups was determined by independent samples t-test and Mann-Whitney U test, as appropriate. Differences between dependent samples were analyzed by paired t-test and Wilcoxon signed-rank test. A p<0.05 was considered sufficient to indicate statistical significance when testing two-sided hypotheses.

## RESULTS

**Fig.1:**
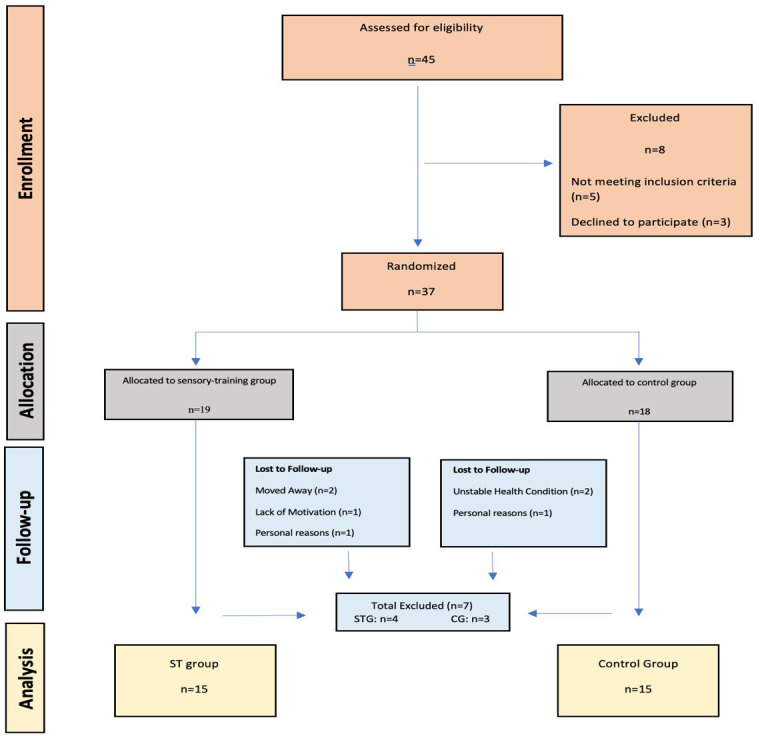
Consort flow diagram of the study.

Forty-five SS who applied for treatment at our university’s physical therapy and rehabilitation clinic were interviewed. Patients were randomized by an independent person. The flow chart of the study is shown in Fig.1. The mean ages of the patients are given in Table-I, and their demographic information is given in Table-II.

**Table-I:** Mean age of SS.

Age		*n*	*Mean±SD*	*Min-Max*	*Med*
	STG	15	59.07±12.73	29-78	62
	CG	15	56.53±13.80	25-75	61
p			0.61		

**Table-II:** Demographics of SS.

		STG (n=15)		CG (n=15)	
		n	%	n	%
Gender	Male	8	53.3	9	40.0
	Female	7	46.7	6	60.0
Hemiplegic Side	R	11	73.3	8	53.3
	L	4	26.7	7	46.7
Dominant Hand	R	13	86.7	13	86.7
	L	2	13.3	2	13.3
Disease Duration	6-12 month	9	60.0	3	20.0
	1-2 year	4	26.6	6	40.0
	2-6 year	1	6.7	4	26.7
	6-10 year	1	6.7	2	13.3
	10+ year	0	0.0	0	0.0

Before treatment, both groups were similar in all sensory functions (p>0.05, Table-III). Pre- and post-treatment evaluations of JTHFT, MFS, GAS, COPM, and SS-QOL are presented in Tables IV–VIII, with no significant pre-treatment differences between groups (p>0.05).

**Table-III:** Sensory Evaluations of SS.

		Decreased n (%)	Normal n (%)
Thumb Localization Test *	STG (n=15)	12 (80.0)	3 (20.0)
	CG (n=15)	12 (80.0)	3 (20.0)
	p	0.59	
Finger Swipe Test **	STG (n=15)	12 (80.0)	3 (20.0)
	CG (n=15)	11 (77.3)	4 (26.7)
	p	0.64	
Stereognozi**	STG (n=15)	9 (60.0)	6 (40.0)
	CG (n=15)	10 (66.7)	5 (33.3)
	p	0.73	

p:Mann-Whitney U Test

* 3 or 6 correct answers “reduced” in 6 repetitions: 4-6 correct answers are “normal”

** Recognizing 7-12 objects is “normal”: recognizing 7 or less objects are “decreased”.

**Table-IV:** Results of JTHFT.

	STG	CG	p*
BTTurning cards	40.14 (18.53-52.65)	38.87 (25.17-49.1)	0.87
ATTurning cards	28.01 (10.36-34.73)	37.43 (24.42-47.9)	**0.02**
p**	<**0.01**	<**0.01**	
BTMoving small objects	47.5 (22.87-59.49)	49.53 (38.2-57.49)	0.71
AT Moving small objects	34.24 (12.97-39.6)	48.7 (35.96-55.03)	**0.01**
p**	**<0.01**	**<0.01**	
BTSimulated feeding	40.65 (21.66-50.66)	40.55 (33.83-48)	0.90
AT Simulated feeding	30.12 (14.27-39.62)	38.67 (31.45-47.03)	**0.01**
p**	**<0.01**	**<0.01**	
BTStacking checkers	29.46 (20.09-48.49)	38.84 (25.88-53.99)	0.32
AT Stacking checkers	25.3 (16.56-33.46)	36.12 (23.56-49.8)	**0.04**
p**	**<0.01**	**<0.01**	
BTMoving empty cans	29.56 (17.82-40.6)	36.22 (27.56-43.51)	0.25
AT Moving empty cans	23.18 (11.88-27.28)	35.74 (26.59-40.17)	**<0.01**
p**	**<0.01**	**<0.01**	
BTMoving full cans	33.9 (20.07-39.72)	39.59 (31.9-54.12)	0.13
AT Moving full cans	25.37 (12.72-32.03)	37.59 (28.3-50.54)	**<0.01**
p**	**<0.01**	**<0.01**	

Data were presented as median (p25, p75).

* p value for between groups comparisons (Mann-Whitney U test).

** p value for within groups comparisons (Wilcoxon signed rank test). BT: Before Treatment AT: After Treatment.

Post-treatment assessments revealed significant improvements in JTHFT (p<0.05) (Table-IV), MFS (p<0.05) (Table-V), GAS (p=0.049) (Table-VI), COPM performance (p=0.001) and satisfaction (p=0.006) (Table-VII), favoring the STG (p<0.05), except for the SS-QOL domains of language, thinking, and vision (p>0.05) (Table-VIII). Based on Cekmece C et al.^9^ the daily living activities in which both groups had difficulties identified from COPM data and are shown in Table-IX.

**Table-V:** Results of MFS.

	STG	CG	p*
***MFS1***			
BT Opening the jar lid	3 (3-7)	7 (3-7)	0.10
AT Opening the jar lid	9 (8-10)	6 (4-8)	**0.02**
p**	**<0.01**	**<0.01**	
***MFS2***			
BT Drawing lines with a ruler	5 (4-6)	5 (4-6)	0.35
AT Drawing lines with a ruler	8 (5-9)	4 (3-7)	**<0.01**
p**	**<0.01**	0.65	
***MFS3***			
BT Big cup holding	6 (3-8)	6 (3-8)	0.16
AT Big cup holding	8 (8-10	5 (4-8)	**0.02**
p**	**<0.01**	**0.01**	
***MFS4***			
BT Small cup holding	5 (2-7)	5 (2-7)	0.23
AT Small cup holding	7 (6-9)	5 (3-7)	**0.01**
p**	**<0.01**	**<0.01**	
***MFS5***			
BT Drinking water	4 (3-5)	4 (3-5)	0.79
AT Drinking water	7 (5-9)	5 (2-7)	**0.01**
p**	**<0.01**	0.24	
***MFS6***			
BT 3 pegs install	4 (2-5)	4 (2-5)	0.98
AT 3 pegs install	6 (4-8)	2 (2-5)	**<0.01**
p**	**<0.01**	0.19	
***MFS7***			
BT Hair combing	4 (3-7)	3 (2-5)	0.08
AT Hair combing	5 (4-9)	4 (3-7)	**0.02**
p**	**<0.01**	**<0.01**	
***MFS8***			
BT Squeezing toothpaste	5 (4-7)	3 (3-5)	0.10
AT Squeezing toothpaste	8 (5-9)	4 (4-6)	**0.01**
p**	**<0.01**	**<0.01**	
***MFS9***			
BT Using a fork and knife	4 (3-7)	3 (2-6)	0.51
AT Using a fork and knife	7 (5-9)	5 (3-7)	**0.02**
p**	**<0.01**	**<0.01**	
***MFS10***			
BT Using a broom	5 (2-6)	4 (2-6)	0.96
AT Using a broom	8 (6-9)	5 (3-7)	**0.03**
p**	**<0.01**	**<0.01**	

**Table-VI:** Results of GAS.

		Before Treatment (Mean±SD)	After Treatment (Mean±SD)	p**
GAS (-2 / +2)	STG	-1.00±0.00	0,73±0,80	<0.01
	CG	-1.00±0.00	0.07±0.96	<0.01
	p*	1.00	0.04	

**Table-VII:** Results of COPM.

	STG	CG	p*
BT Performance	3.2 (2.8-3.6)	3.8 (3.2-4)	0.18
AT Performance	5.6 (5 - 6.8)	4 (4 - 5)	**<0.01**
p**	**<0.01**	**<0.01**	
BT Satisfaction	4 (3.2-4.6)	4 (3-5)	0.80
AT Satisfaction	6.2 (5.6 - 7)	5 (4.2 - 6)	**<0.01**
p**	**<0.01**	**<0.01**	
BT Total	7.2 (6.6 - 7.8)	7.6 (6.4 - 9)	0.57
AT Total	12.2 (10.6 - 13)	9.8 (8.2 - 11)	**<0.01**
p**	**<0.01**	**<0.01**	

**Table-VIII:** Results of SSQLS.

	STG p	CG	p*
BT Energy	6 (4-7)	5 (3-8)	0.51
AT Energy	10 (8-14)	7 (5-10)	**0.02**
p**	**<0.01**	**0.01**	
BT Family Roles	5 (5-8)	5 (4-5)	0.14
AT Family Roles	8 (7-11)	7 (5-8)	**0.02**
p**	**<0.01**	**<0.01**	
BT Language	15 (10-25)	15 (6-25)	0.93
AT Language	20 (13-25)	19 (10-25)	0.74
p**	**<0.01**	**0.01**	
BT Mobility	12 (9-16)	14 (9-23)	0.22
AT Mobility	23 (19-26)	15 (11-23)	**0.02**
p**	**<0.01**	0.08	
BT Mood	15 (10-19)	12 (10-18)	0.53
AT Mood	20 (18-24)	15 (13-20)	**<0.01**
p**	**<0.01**	**<0.01**	
BT Personality	7 (4-12)	6 (3-12)	0.41
AT Personality	11 (8-15)	8 (6-13)	**0.03**
p**	**<0.01**	**<0.01**	
BT Self-Care	15 (11-17)	14 (11-17)	0.53
AT Self-Care	20 (18-24)	16 (13-17)	**<0.01**
p**	**<0.01**	**<0.01**	
BT Social Roles	9 (7-9)	7 (5-9)	0.07
AT Social Roles	13 (12-15)	10 (8-13)	**<0.01**
p**	**<0.01**	**<0.01**	
BT Thinking	6 (4-9)	6 (3-13)	0.75
AT Thinking	10 (8-15)	10 (5-14)	0.16
p**	**<0.01**	**<0.01**	
BT Upper Extremity Function	12 (7-15)	11 (9-14)	0.96
AT Upper Extremity Function	19 (13-23)	14 (10-16)	**0.01**
p**	**<0.01**	**<0.01**	
BT Seeing	11 (9-15)	15 (12-15)	0.28
AT Seeing	13 (11-15)	15 (12-15)	0.19
p**	0.08	0.06	
BT Work/ Productivity	5 (3-6)	5 (4-6)	0.93
AT Work/ Productivity	8 (6-11)	6 (4-8)	**0.03**
p**	**<0.01**	**0.03**	
BT Total	115 (104-159)	118 (97-141)	0.77
AT Total	174 (163-216)	148 (120-154)	**<0.01**
p**	**<0.01**	**<0.01**	

**Table-IX:** Activities in which participants have problems according to COPM.

		STG (n=15)		CG (n=15)	
		n	%	n	%
Self-Care Activities	Brushing teeth	3	20	4	26.6
	Shaving	4	26.6	1	6.6
	Take a shower	7	46.6	9	60
	Cut nail	4	26.6	2	13.3
	Dressing-Undressing	5	33.3	4	26.6
Manufacturer Activities	Cooking	5	33.3	3	20
	Go to work	3	20	4	26.6
	Cleaning up	4	26.6	5	33.3
Free Time Activities	Take a walk	5	33.3	3	20
	Spending time with grandchildren	3	20	4	26.6
	Spending time with family	3	20	4	26.6
	Knit	2	13.3	1	6.6
	Visiting relatives/friends	3	20	4	26.6

## DISCUSSION

In this randomized controlled trial, we investigated the effect of sensory therapy on upper extremity function, ADL, and quality of life in SS. After a three-week intervention, we observed significant improvements in upper extremity function, ADL performance and satisfaction, and quality of life in SS receiving sensory therapy compared to those receiving traditional treatments without sensory training. There are very few studies in the literature evaluating the contribution of sensory training to motor function, activity, and participation in SS.^10^-^12^ Furthermore, this study is important as it is the first study conducted in our country.

Our study used the JTHFT to evaluate plegic upper extremity motor function.^13^ Proprioceptive impairments in SS often hinder coordinated movements involving grip, reach, and shoulder stabilization. The greater improvement in these activities in the STG supports that additional sensory therapy enhances upper extremity functionality in ADL. This finding aligns with previous studies reporting positive effects of sensory rehabilitation on motor function.^14^-^16^

Published studies examining the effectiveness of sensory therapies on are limited.^10^-^12^,^17^ In the present study, we used MFS and GAS scales to evaluate the ADLs of SS.^18^,^19^ In the present study, both the MFS and GAS scores showed a significant difference in favor of the STG. However, 8/15 SS in the STG and 4/15 SS in the control group (CG) reached the expected level, and 2 SS in the STG and one stroke survivor in the CG exceeded their expected goals. Studies have reported that sensory therapies applied to SS lead to significant improvements not only in balance, upper extremity use and motor functions, but also in ADL performance.^12^,^17^,^20^

Published studies on the effectiveness of sensory therapies are limited.^10^–^12^,^17^ In this study, MFS and GAS scales were used to assess ADLs of SS.^18^,^19^ Both scores showed significant improvement in favor of the STG. Moreover, more participants in the STG reached or exceeded their expected goals. These findings align with previous studies reporting that sensory therapies improve not only balance, upper extremity use, and motor functions but also ADL performance.^12^,^17^,^20^

The COPM was used to assess activity and participation. Post-treatment scores significantly favored the STG, as is consistent with the literature.^10^ An improvement of ≥2 points on COPM indicates a clinically meaningful change,^21^,^22^ and in this study, the STG improved by 2.34 points in performance and 2.45 in satisfaction, whereas the CG improved by 0.7 and 1.2 points, respectively. This suggests that patient-centered ADL education combined with sensory therapy, effectively enhances functional independence.

Stroke is one of the leading causes of activity and participation limitations that negatively affect health-related quality of life.^23^,^24^ To examine how stroke affects the quality of life of SS and the effect of sensory therapy, we used SS-QOL in this study. When the results of the ST and CG were compared after treatment, there was a significant difference in favor of the STG in all areas except for the “Language”, “Thinking”, and “Sight” domains. Although there are studies examining the effectiveness of sensory therapy in SS, there is no study evaluating the effect of sensory therapy on the quality of life of SS.

The major strengths of this study are its randomised controlled design, blinded assessments, and use of multiple validated outcome measures. These elements all enhance reliability and reduce bias. Another strength of this study is that there are limited studies in the literature on the effectiveness of intensified sensory therapies applied in chronic SS, so this study with a randomized controlled design fills an important information gap. The results of the present study showed that sensory training has positive effects on upper extremity functionality and independence, participation in ADL, and thus improved quality of life in SS. Despite these strengths, larger sample sizes and longer follow-up studies are needed to generalize the findings.

### Limitations

The first limitation of this study was the duration of treatment. Another limitation is that this study coincided with the Covid-19 pandemic. The pandemic prevented us from increasing the number of inpatients and outpatients and patient diversity.

## CONCLUSIONS

The study showed that sensory therapies applied with conventional treatments increased upper extremity functions, ADL performance, participation rate and quality of life of the SS. Our findings are of great importance as it is one of the few studies conducted on this subject. Based on our findings, it is recommended that sensory training be incorporated into rehabilitation protocols at the early stages to support upper extremity function and improve participation and quality of life in individuals with SS.
